# Human Infections with *Rickettsia raoultii*, China

**DOI:** 10.3201/eid2005.130995

**Published:** 2014-05

**Authors:** Na Jia, Yuan-Chun Zheng, Lan Ma, Qiu-Bo Huo, Xue-Bing Ni, Bao-Gui Jiang, Yan-Li Chu, Rui-Ruo Jiang, Jia-Fu Jiang, Wu-Chun Cao

**Affiliations:** Beijing Institute of Microbiology and Epidemiology, Beijing, China (N. Jia, L. Ma, W.-C. Cao);; Mudanjiang Forestry Central Hospital, Mudanjiang, China (Y.-C. Zheng, Q.-B. Huo, Y.-L. Chu);; State Key Laboratory of Pathogens and Biosecurity, Beijing (X.-B. Ni, B.-G. Jiang, R.-R. Jiang, J.-F. Jiang)

**Keywords:** Rickettsia raoultii, rickettsia, human infections, ticks, Dermacentor silvarum, vector-borne infections, China

## Abstract

We used molecular methods to identify *Rickettsia raoultii* infections in 2 persons in China. These persons had localized rashes around sites of tick bites. *R. raoultii* DNA was detected in 4% of *Dermacentor silvarum* ticks collected in the same area of China and in 1 feeding tick detached from 1 patient.

*Rickettsia raoultii* was first detected in *Rhipicephalus pumilio* and *Dermacentor nuttalli* ticks from the former Soviet Union in 1999 and initially named RpA4, DnS14, and DnS28 (*1*). In 2008, it was identified as a novel *Rickettsia* species on the basis of its genetic and serologic characteristics (*2*). Since its emergence, *R*. *raoultii* has been found to be associated with *Dermacentor* ticks throughout Europe (*3*) and in some parts of Asia, including Mongolia (*4*) and China (*5*,*6*). 

A *D*. *marginatus* tick detached from a patient in France with tick-borne lymphadenopathy/*Dermacentor* tick–borne necrosis erythema and lymphadenopathy was positive for *R*. *raoultii*, suggesting its pathogenicity for humans (*2*). Seven additional *R*. *raoultii*–infected cases were later diagnosed in France (*7*). We report 2 cases of *R*. *raoultii* infection in northeastern China.

## The Study

On May 15, 2012, a 67-year-old man came to Mudanjiang Forestry Central Hospital in Mudanjiang, China, with an attached tick on his left shoulder. The feeding tick was detached in the outpatient department and subsequently identified as *D. silvarum* by an entomologist. Four days later, a painful erythematous rash developed around the site of the tick bite, and the patient returned to the hospital for treatment. The rash was irregular, round, and ≈1.5 cm in diameter. No other symptoms and signs were found.

On May 21, 2012, a 30-year-old man came to the same hospital with asthenia, anorexia, nausea, and a painful rash on his abdomen. He had been bitten by a tick 4 days before onset of symptoms. Physical examination identified a 20 × 5 cm erythematous rash on his abdomen around a tick-bite eschar. Vesicles were seen surrounding the site of the tick bite. Lymphadenopathy was not observed.

Routine laboratory and hematologic assays for blood and serum samples from both patients showed standard results. Because rashes were found during physical examinations and infection with spotted fever group rickettsia was suspected, oral doxycycline (100 mg, twice a day for 2 weeks) was then prescribed. The infections of the 2 patients were successfully treated at home and did not recur.

DNA was extracted from anticoagulated blood samples by using a Blood DNA Extraction Kit (QIAGEN, Germantown, MD, USA) according to the manufacturer’s instructions. PCRs specific for the conserved citrate synthase gene (*glt*A) and spotted fever group–restricted outer membrane protein A gene (*omp*A) were conducted as described (*8*) and followed by sequencing. Both partial *gltA* (341 bp) and *ompA* (325 bp) sequences from the 2 patients were identical to each other and to those of *R. raoultii* (Figure).

**Figure:**
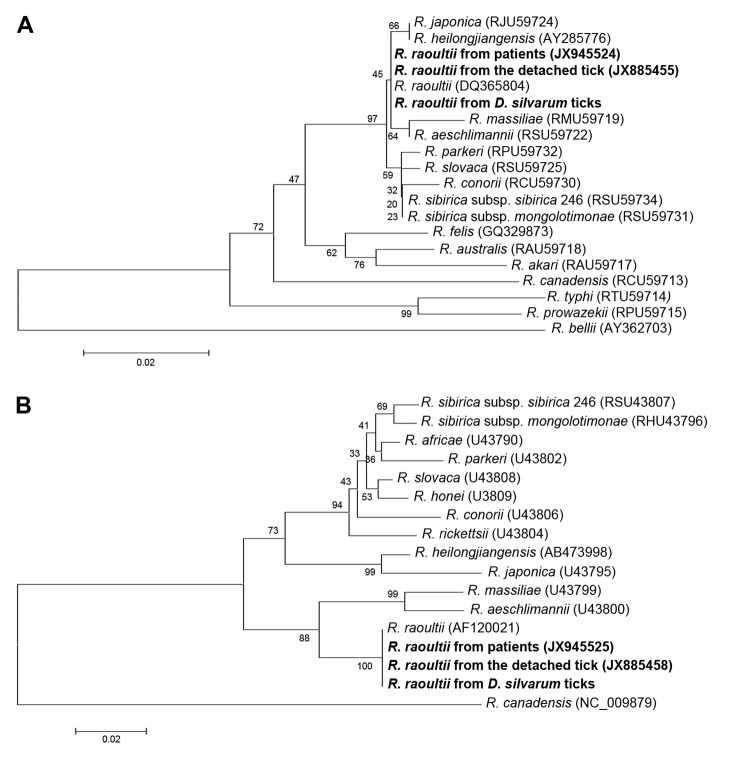
Phylogenetic analysis of spotted fever group *Rickettsia* species, China, based on A) partial (341 bp) citrate synthase gene and B) partial (325 bp) 190-kDa outer membrane protein gene. Trees were obtained by using the neighbor-joining method, distances were calculated by using Kimura 2-parameter analysis, and analysis was conducted by using Mega 5.0 software (www.megasoftware.net/). Nucleotide sequences determined in this study are indicated in boldface. Percentage of replicate trees in which associated taxa clustered in the bootstrap test (1,000 replicates) is shown to the left of each branch. Trees are drawn to scale, and branch lengths have the same units as evolutionary distances used to infer the phylogenetic tree. GenBank accession numbers of sequences used in phylogenetic analysis are indicated in parentheses. Scale bars indicate nucleotide substitutions per site.

PCR results for other tick-borne pathogens, including *Anaplasma phagocytophilum*, *Ehrlichia chaffeensis, Babesia* spp., *Francisella tularensis*, and *Borrelia burgorferi* sensu lato, were negative. Serum specimens were tested by indirect immunofluorescence assay for IgM and IgG against *R. heilongjiangensis*, which was a known *Rickettsia* species circulating in the study area (*9*). IgM titers of 64 and 128, respectively, were observed in serum samples obtained 3 days after disease onset from both patients. The IgG titer was 64 for both patients.

A feeding *D. silvarum* tick detached from the first patient was positive for rickettsial *glt*A and *omp*A by PCR. Sequences were identical to those amplified from the 2 patients (Figure). We used primers CS2d-CSend and Rr190.70-Rr190.602 to amplify longer fragments of *glt*A (1,150 bp) and *omp*A (530 bp), respectively (*8*). Analyses showed that these sequences were identical to corresponding sequences of *R*. *raoultii* identified in a tick detached from a French patient (*2*).

To identify local natural foci, host-seeking ticks were collected by dragging a fabric flag over vegetation in the area where the 2 patients lived. An entomologist identified all ticks by morphologic features to the species level and developmental stage. DNA was extracted from ticks by using a Tissue DNA Extract Kit (Tiangen Biotechnique Inc., Beijing, China) following the manufacturer’s instructions. PCR was performed to amplify rickettsial *glt*A and *omp*A fragments, and sequencing was performed (*8*). *R. raoultii* was identified in 3 (4%) of 75 adult *D. silvarum* ticks and 0 of 453 *Ixodes persulcatus* ticks tested (Figure).

## Conclusions

Eight cases of human infection with *R. raoultii* have been reported in France. All patients had tick-borne lymphadenopathy/*Dermacentor* tick–borne necrosis erythema and lymphadenopathy, which was defined as the association of a tick bite, an inoculation eschar on the scalp, and cervical lymphadenopathies (*10*). 

Although the 2 patients in China had only painful rashes around the sites of tick bites, rather than tick-borne lymphadenopathy/*Dermacentor* tick–borne necrosis erythema and lymphadenopathy, the difference in clinical features might be the result of location of tick bites. Tick-borne lymphadenopathy/*Dermacentor* tick–borne necrosis erythema and lymphadenopathy is usually observed in patients after tick bites on the scalp, regardless of whether the disease is caused by *R. slovaca*, *R. rioja*, or *R. raoultii* (*2*,*7*,*11*). The 2 patients had tick bites on abdomen and shoulder, respectively, but no cervical lymphadenopathy developed.

Isolation in cell cultures is the ultimate diagnostic method for rickettsial infections, but it is available only in biosafety level 3 laboratories (*9*). However, serologic tests are useful tools, and indirect immunofluorescence assay is considered the reference method. PCR, followed by sequencing is mainly used to detect and identify *Rickettsia* species and is also an effective diagnostic method (*9*,*12*).

Of the 8 cases of human infection with *R. raoultii* in France, 4 were identified by PCR amplification from ticks detached from patients, and 4 were identified by rickettsial-specific serologic assays (*2*,*7*). In contrast, we identified 2 cases of human infection with *R. raoultii* in China by molecular detection and sequence determination of the pathogen in blood samples and from a tick detached from 1 of the patients. Although only acute-phase (3 days after disease onset) serum samples were available, the presence of IgM and IgG against *R. heilongjiangensis* supported the diagnoses (*12*).

*R. raoultii* has been detected in *Dermacentor* spp. ticks, such as *D. silvarum, D. nuttalli, D. reticulatus*, and* D. marginatus* (*1*,*2*), and infection rates in Europe range from 2% to 80% (*13*). *R. raoultii* was also found in 4% of *D. silvarum* ticks collected in the same area of China. We previously identified a novel rickettsial agent identical to DnS14 from *D. silvarum* ticks, which was later named *R. raoultii*, in Jilin Province neighboring this study site (*6*). *D. silvarum* ticks are distributed over a wide area in northeastern China, parasitize many domestic and wild animals, and readily feed on humans as alternate hosts. Extended investigation and tick surveillance are required to understand the distribution of *R. raoultii* in this region.

The current recommended therapeutic regimen for rickettsiosis is administration of tetracyclines; doxycycline is the preferred agent because of its dosage (twice a day) and better patient tolerance. The 2 patients received doxycycline treatment at home and had uneventful recoveries. These findings indicate that doxycycline should be administrated as soon as possible to patients who have been exposed to ticks and have clinical manifestations suggestive of rickettsiosis.

## References

[1] Rydkina E, Roux V, Fetisova N, Rudakov N, Gafarova M, Tarasevich I, New rickettsiae in ticks collected in territories of the former Soviet Union. Emerg Infect Dis. 1999;5:811–4 . 10.3201/eid0506.990612 10603217 PMC2640811

[2] Mediannikov O, Matsumoto K, Samoylenko I, Drancourt M, Roux V, Rydkina E, *Rickettsia raoultii* sp. nov., a spotted fever group rickettsia associated with *Dermacentor* ticks in Europe and Russia. Int J Syst Evol Microbiol. 2008;58:1635–9. 10.1099/ijs.0.64952-0 18599708

[3] Spitalská E, Stefanidesova K, Kocianova E, Boldis V. *Rickettsia slovaca* and *Rickettsia raoultii* in *Dermacentor marginatus* and *Dermacentor reticulatus* ticks from Slovak Republic. Exp Appl Acarol. 2012;57:189–97. 10.1007/s10493-012-9539-8 22392435

[4] Speck S, Derschuma H, Damdindorj T, Dashdavaa O, Jiang J, Kaysser PB, *Rickettsia raoultii,* the predominant *Rickettsia* found in Mongolian *Dermacentor nuttalli.* Ticks Tick Borne Dis. 2012;3:227–31. 10.1016/j.ttbdis.2012.04.001 22784401

[5] Tian ZC, Liu GY, Shen H, Xie JR, Luo J, Tian MY. First report on the occurrence of *Rickettsia slovaca* and *Rickettsia raoultii* in *Dermacentor silvarum* in China. Parasit Vectors. 2012;5:19. 10.1186/1756-3305-5-19 PMC3292836 22257726

[6] Cao WC, Zhan L, De Vals SJ, Wen BH, Yang H, Richardus JH, Molecular detection of spotted fever group *Rickettsia* in *Dermacentor silvarum* from a forest area of northeastern China. J Med Entomol. 2008;45:741–4. 10.1603/0022-2585(2008)45[741:MDOSFG]2.0.CO;2 18714877

[7] Parola P, Rovery C, Rolain JM, Brouqui P, Davoust B, Raoult D. *Rickettsia slovaca* and *R. raoultii* in tick-borne rickettsioses. Emerg Infect Dis. 2009;15:1105–8. 10.3201/eid1507.081449 19624931 PMC2744242

[8] Jia N, Zheng YC, Jiang JF, Ma L, Cao WC. Human infection with *Candidatus Rickettsia tarasevichiae.* N Engl J Med. 2013;369:1178–80. 10.1056/NEJMc1303004 24047080

[9] La Scola B, Raoult D. Laboratory diagnosis of rickettsioses: current approaches to the diagnosis of old and new rickettsial diseases. J Clin Microbiol. 1997;35:2715–27 . 9350721 10.1128/jcm.35.11.2715-2727.1997 PMC230049

[10] Raoult D, Lakos A, Fenollar F, Beytout J, Brouqui P, Fournier PE. Spotless rickettsiosis caused by *Rickettsia slovaca* and associated with *Dermacentor* ticks. Clin Infect Dis. 2002;34:1331–6. 10.1086/340100 11981728

[11] Oteo JA, Portillo A. Tick-borne rickettsioses in Europe. Ticks Tick Borne Dis. 2012;3:271–8. 10.1016/j.ttbdis.2012.10.035 23177355

[12] Centers for Disease Control and Prevention. Fatal cases of Rocky Mountain spotted fever in family clusters—three states, 2003. MMWR Morb Mortal Wkly Rep. 2004;53:407–10 . 15152183

[13] Silaghi C, Dietmar H, Thiel C, Pfister K, Pfeffer M. Spotted fever group rickettsiae in ticks, Germany. Emerg Infect Dis. 2011;17:890–2. 10.3201/eid1705.101445 21529404 PMC3321775

